# Free IL-18 in NLRC4-associated autoinflammatory disease without macrophage activation syndrome

**DOI:** 10.1093/rheumatology/keag363

**Published:** 2026-07-13

**Authors:** Mathijs Willemsen, Thea H M Schoonbrood, Emiliana Rodriguez, Willem Roosens, Ellen De Langhe, Rik Schrijvers, Cem Gabay

**Affiliations:** Department of Rheumatology, Maastricht University Medical Centre, Maastricht, The Netherlands; Department of Rheumatology, Maastricht University Medical Centre, Maastricht, The Netherlands; Division of Rheumatology, Department of Medicine, Geneva University Hospitals, Geneva, Switzerland; Department of Pathology and Immunology, Faculty of Medicine, University of Geneva, Geneva, Switzerland; Geneva Centre for Inflammation Research, Geneva, Switzerland; Allergy and Clinical Immunology Research Group, Department of Microbiology, Immunology and Transplantation, KU Leuven, Leuven, Belgium; Department of Rheumatology, University Hospitals Leuven, Leuven, Belgium; Allergy and Clinical Immunology Research Group, Department of Microbiology, Immunology and Transplantation, KU Leuven, Leuven, Belgium; Division of Allergy and Clinical Immunology, Department of General Internal Medicine, University Hospitals Leuven, Leuven, Belgium; Division of Rheumatology, Department of Medicine, Geneva University Hospitals, Geneva, Switzerland; Department of Pathology and Immunology, Faculty of Medicine, University of Geneva, Geneva, Switzerland; Geneva Centre for Inflammation Research, Geneva, Switzerland

**Keywords:** NLRC4, macrophage activation syndrome, IL-18, autoinflammatory disease

## Abstract

**Objectives:**

NLRC4-associated autoinflammatory diseases (NLRC4-AID) cover a clinical spectrum from relatively mild familial cold autoinflammatory syndrome 4 (FCAS4) to life-threatening macrophage activation syndrome (MAS). Free IL-18 has been shown to pathogenically promote MAS, but the pathological relevance of free IL-18 in NLRC4-AID without MAS has not been explored. We investigated free IL-18 and related cytokines in five germline p.S445P and one somatic p.V341L NLRC4-AID patients without MAS.

**Methods:**

Clinical, genetic and laboratory data, as well as serum samples were obtained from NLRC4-AID patients without MAS. Human IL-6, soluble CD25, CXCL9, CXCL10, IL-12p70, IFN-γ, total IL-18, IL-1β, TNF-α and IL-1Ra were assessed using a bead-based assay. Free IL-18 was assessed using a proprietary ELISA.

**Results:**

Total and free IL-18 were elevated both in the FCAS4 patients and the NLRC4-AID patient harbouring a heterozygous somatic NLRC4 variant restricted to the haematopoietic compartment. Despite the association between free IL-18 and MAS, we found no relevant correlation between total and free IL-18 and laboratory markers of inflammation or MAS. Accordingly, MAS-associated cytokines IFN-γ, CXCL9 and CXCL10 were not elevated in NLRC4-AID patients without MAS.

**Conclusion:**

Germline and somatic NLRC4-AID without MAS are associated with elevated levels of both total and free IL-18. Our findings in the somatic NLRC4-AID patient suggest that free IL-18 originating from the haematopoietic system alone is not sufficient to drive MAS. Our findings indicate that IL-18 is necessary but not sufficient to drive MAS and suggest a pathological role of IL-18 beyond MAS in NLRC4-AID.

Rheumatology key messages

- Germline and somatic NLRC4-AID without MAS are associated with detectable free IL-18.
- IL-18 originating from the haematopoietic system seems to be insufficient to drive MAS

## Introduction

Gain-of-function variants in *NLRC4* were first described in 2014 [1, 2] to cause a syndrome of autoinflammation with infantile enterocolitis with exceptional susceptibility to macrophage activation syndrome (MAS). Soon after, patients with urticarial rash, arthritis and fever were also reported to harbour *NLRC4* variants [3], a syndrome coined familial cold autoinflammatory syndrome 4 (FCAS4), due to its similarity to *NLRP3*-associated FCAS1. Nowadays, >25 *NLRC4* variants have been described and the array of clinical manifestations has expanded considerably to include neonatal-onset multisystem inflammatory disease and SLE-like disease. Collectively, this clinical spectrum has been termed NLRC4-associated autoinflammatory diseases (NLRC4-AID) [4, 5].

Under physiological conditions, the recognition of specific bacterial proteins in the cytosol triggers the assembly of the NLRC4 inflammasome. The subsequent caspase-1-dependent proteolytic cleavage of pro-IL-1β, pro-IL-18 and gasdermin D causes pyroptosis, a form of inflammatory cell death, associated with the release of mature IL-1β, IL-18 and several intracellular alarmins into the extracellular space. This innate effector mechanism serves as a powerful protective inflammatory response against intracellular bacterial pathogens, especially at barrier tissues [5, 6].

Although other inflammasomes also process pro-IL-18 to its bioactive form, NLRC4-AID, for unknown reasons, distinguishes itself from other inflammasomopathies by chronically elevated IL-18 levels and, in more severe cases, susceptibility to MAS. Once released, IL-18 is quickly neutralized by its naturally occurring antagonist IL-18 binding protein (IL-18BP), and only extremely high levels of total IL-18 result in detectable bioactive free IL-18 [5]. The presence of free IL-18 was recently shown to define a group of autoinflammatory diseases, including NLRC4-AID, at risk for MAS [5, 7]. Interestingly, both germline [8–10] and somatic [11] NLRC4-AID patients without MAS also display elevated total IL-18 levels, but free IL-18, and thus the pathological relevance of IL-18, has never been investigated in these groups. In this report, we sought to determine IL-18 and related cytokines in a kindred with FCAS4 and a somatic NLRC4 patient.

## Methods

### Participant recruitment

FCAS4 patients treated at Maastricht University Medical Centre were recruited as part of a retrospective cohort study which inventories monogenic autoinflammatory diseases in the Euregion Meuse-Rhine. The clinical course of one FCAS4 patient was previously published [10]. Disease controls without systemic inflammation (non-inflammatory controls (NIC, *n* = 11)) were recruited under the same protocol from the outpatient neurology, rheumatology and internal medicine clinics at Maastricht University Medical Centre. The Maastricht University Medical Centre ethics committee approved this study (2024–0211-A-3).

The somatic NLRC4 patient was recruited at University Hospitals Leuven as part of the somatic mosaicism in immune disorders (SMID) study. This protocol was approved by the University Hospitals Leuven ethics committee (s67807). All participants provided written informed consent.

**Figure 1:**
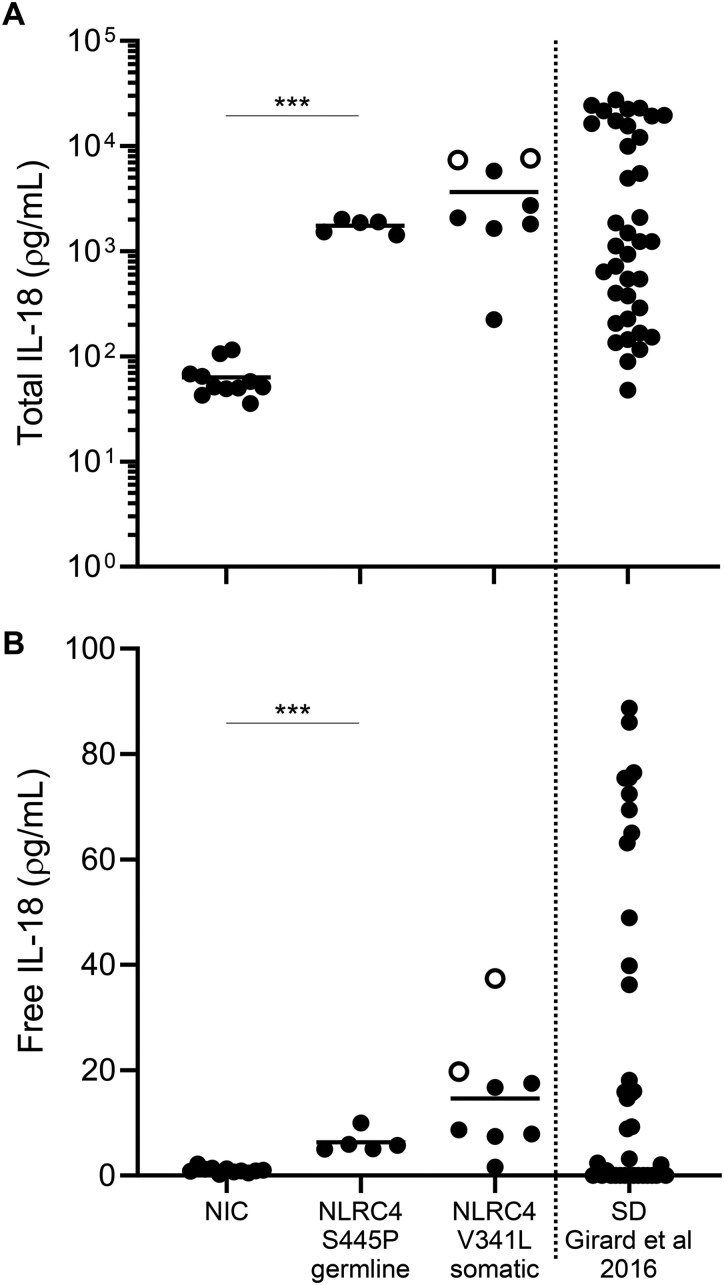
Total and free IL-18 in NLRC4-AID without MAS. Levels of total (**A**) and free (**B**) IL-18 in NIC, FCAS4, somatic NLRC4-AID patient and SD [12]. ***P > 0.001. For the somatic NLRC4-AID patient, open circles depict samples collected during treatment with canakinumab

Total and free IL-18 levels of 37 adult-onset Still’s disease (SD) patients which were previously published in the *Journal* are included in Fig. 1 to allow direct comparison to the NLRC4-AID patients [12]. These data were generated in the same lab using the same assays as described below.

### Cytokine measurements

Free human IL-18 was measured in undiluted serum by sandwich ELISA using proprietary recombinant human IL-18BPa (AB2 Bio, Lausanne, Switzerland) as capture protein, as previously described [12]. Human IL-6, soluble CD25 (sCD25, also known as soluble IL-2 receptor), CXCL9, CXCL10, IL-12p70, IFN-γ, total IL-18, IL-1β, TNF-α and IL-1Ra were measured in undiluted or 5-fold diluted serum using a BioLegend LEGENDplex™ Custom Panel, according to the manufacturer’s instructions.

### Statistical analysis

NIC and FCAS4 patients were compared by Mann–Whitney test. When multiple samples were available for a FCAS4 patient, the mean value of all available samples per patient was used for statistical analysis. Statistical analysis was not performed on longitudinal samples of the somatic NLRC4-AID patient as these are not independent observations. Correlations between total and free IL-18 and laboratory markers of inflammation and MAS (i.e. haemoglobin, leukocytes, thrombocytes, ferritin, sCD25, lactate dehydrogenase, ESR and CRP) were assessed using Spearman correlation calculations using eight longitudinal samples from the somatic NLRC4-AID patient, and nine samples from five FCAS4 patients (including four repeated measurements), respectively. Lactate dehydrogenase was excluded from the correlation analysis of FCAS4 patients due to low variability (Supplementary Table S1). All analyses were carried out using GraphPad Prism software (version 9.0; GraphPad Software, Inc., San Diego, CA, USA).

## Results

Five FCAS4 patients harbouring a germline heterozygous p. S445P variant and one patient harbouring a somatic heterozygous p.V341L variant restricted to the haematopoietic compartment [11] were included. Historical samples could be retrieved from four FCAS4 patients (total *n* = 9 samples for five FCAS4 patients). Four of five FCAS4 patients were previously treated with anakinra, but it was stopped in three out of four due to injection-site reactions. One patient was never treated with anakinra due to a satisfactory response to etanercept [10]. At the time of sampling, three of five FCAS4 patients were treated with etanercept, one patient with anakinra and one patient was on a self-requested drug holiday (Supplementary Table S1). Eight historical samples were collected from the somatic NLRC4-AID patient, of which two during treatment with canakinumab (Supplementary Table S2).

As previously reported [8–10], total IL-18 was significantly higher in FCAS4 patients compared with NIC. All FCAS4 samples exceeded NIC levels. Total IL-18 levels of the NLRC4-AID patients were comparable to SD patients, although some SD patients showed highly elevated total levels of IL-18 (Fig. 1A). Total IL-18 levels were sufficient to overwhelm endogenous antagonism by IL-18BP as evidenced by detectable free IL-18 in all FCAS4 patients. Levels of free IL-18 were significantly higher compared with NIC, in which free IL-18 was barely detectable (Fig. 1B). Levels of free IL-18 were comparable to SD patients, although the same SD patients with highly elevated total IL-18 also displayed highly elevated free IL-18 (Fig. 1B). It is noteworthy that none of the SD patients had MAS at the time of sampling [12]. Total and free IL-18 were also higher than NIC levels in all eight longitudinal samples of the somatic NLRC4-AID patient (Fig. 1A and B).

**Figure 2:**
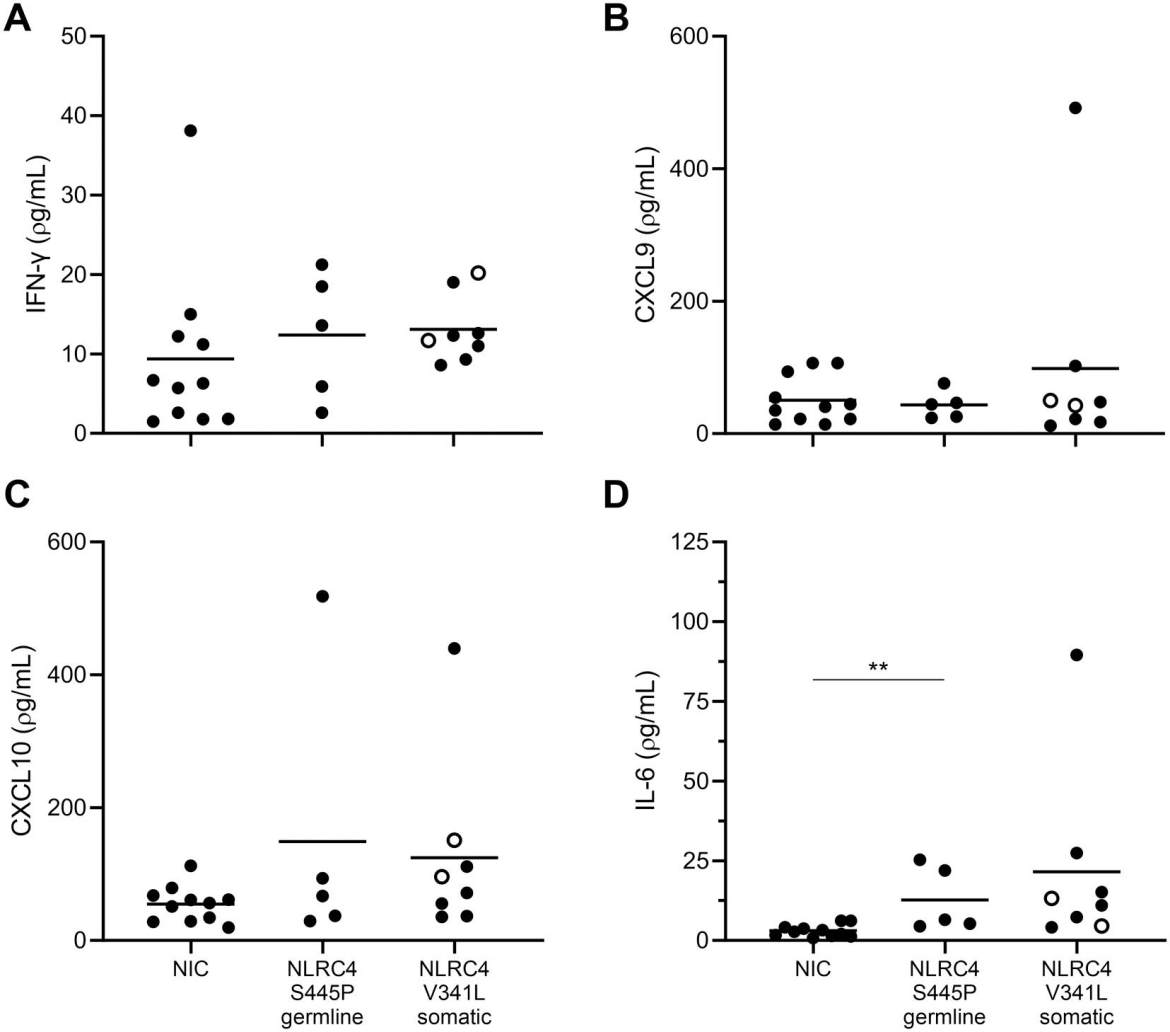
Cytokine levels in NLRC4-AID without MAS. Levels of IFN-γ (**A**), CXCL9 (**B**), CXCL10 (**C**), IL-6 (**D**) in NIC, FCAS4 and a somatic NLRC4-AID patient. **P > 0.01. For the somatic NLRC4-AID patient, open circles depict samples collected during treatment with canakinumab

As previous work suggests that detectable free IL-18 is restricted to a subset of autoinflammatory diseases at risk of MAS [7], we assessed IFN-γ and IFN-γ-induced chemokines CXCL9 and CXCL10 as these are highly elevated in NLRC4-AID MAS [7, 13, 14]. Of note, none of the FCAS4 patients fulfilled any of the HLH-2004 or MAS-2016 criteria at the time of sampling (Supplementary Table S1). Also, we did not find a correlation between total or free IL-18 and laboratory markers of inflammation or MAS when analysing longitudinal samples of the somatic NLRC4-AID patient (Supplementary Fig. S1A and S2, Supplementary Table S2). We did identify a significant positive correlation between free IL-18 and leucocyte count in FCAS4 patients (Supplementary Fig. S1B). This correlation is unlikely to be due to MAS as during active MAS high free IL-18 levels associate with cytopenias. In accordance with the absence of clinical or laboratory indicators of MAS, levels of IFN-γ, CXCL9 and CXCL10 were not different when comparing FCAS4 patients to NIC (Fig. 2A–C). Longitudinal samples from the somatic NLRC4-AID patient also did not demonstrate a persistent increase in IFN-γ, CXCL9 or CXCL10 (Fig. 2A–C).

In addition to total and free IL-18, IL-6 was also significantly increased in FCAS4 patients compared with NIC (Fig. 2D). Six out of eight longitudinal samples from the somatic NLRC4-AID patient also showed IL-6 levels above NIC levels. Levels of TNF-α, IL-1β, IL-12p70 and IL-1Ra showed no differences (Supplementary Fig. S3A–D).

## Discussion

The study of primary hemophagocytic lymphohistiocytosis (HLH), caused by defective granule-mediated cytotoxicity, has been instrumental in shaping our understanding of MAS, a form of secondary HLH. It is becoming increasingly clear that the various causes of HLH converge on cytotoxic T lymphocyte hyperactivation and IFN-γ overproduction [15]. Extremely high levels of IL-18 associated with detectable free IL-18 are largely restricted to patients at risk of MAS. This includes patients with SD (both juvenile and adult-onset) and monogenic autoinflammatory diseases such as NLRC4-AID [5, 7]. Mechanistically, IL-18 is known to induce IFN-γ production, which potentially explains the relationship between IL-18 and MAS [5, 15]. A pathological role of free IL-18 outside the context of MAS has seldom been explored. The study of NLRC4-AID allows for a unique insight into IL-18 biology, as high total IL-18 levels are associated with a clinical spectrum varying from relatively mild FCAS4 to life-threatening MAS.

In this study, we investigated total and free IL-18 and related cytokines in five germline p.S445P FCAS4 and one somatic haematopoietic-restricted p.V341L NLRC4-AID patients, all without clinical or laboratory markers indicative of MAS. Although free IL-18 elevation was previously shown to be restricted to patients with MAS [7], we detected free IL-18 in all included NLRC4-AID patients and levels were higher than in controls. These findings resemble the recent detection of free IL-18 in *PSTPIP1*-mutated PAPA patients, which are also not at risk of MAS [16]. Additionally, NLRC4-AID MAS patients who survive infancy have in some cases gone decades with persistently elevated total, and presumably free, IL-18 without overt MAS [1, 2, 5]. Altogether, these data support the notion that detectable free IL-18 is insufficient to drive MAS.

The levels of free IL-18 detected in this study were relatively low, and higher levels are generally observed in patients with active MAS, including NLRC4-AID MAS [7, 13, 16]. However, free IL-18 levels in MAS-prone SD patients were similar or even lower than in our NLRC4-AID patients [12]. In addition, levels of free IL-18 in some *PSTPIP1*-mutated PAPA patients and SD patients, both without MAS, showed free IL-18 levels comparable to those observed in fulminant NLRC4-AID MAS [13, 16]. Therefore, it seems unlikely that only quantitative differences in free IL-18 explain the lack of MAS in our NLRC4-AID patients.

Beyond quantitative differences, several qualitative factors may also influence the risk of MAS. For example, the cellular origin of IL-18 could impact pathology. A mouse model suggests that the intestinal epithelium, and not the haematopoietic system, is the source of IL-18 in NLRC4-AID [7]. However, our findings of elevated total and detectable free IL-18 in longitudinal samples of the somatic haematopoietic-restricted p.V341L NLRC4-AID patient provide evidence that the human haematopoietic system can serve as an additional or alternative source. The fact that the p.V341L NLRC4 variant is associated with enterocolitis and MAS in germline state [17], when the variant is also present in the intestinal epithelium, seems to support our previous hypothesis that lack of NLRC4 gain-of-function in the gut contributes to the absence of MAS [4]. This hypothesis is further supported by mouse transcriptional data that show constitutive co-expression of *Nlrc4* and *Il18* in epithelial tissues, which uniquely positions NLRC4 to mobilize the large epithelial reservoir of pro-IL-18 [7]. Quantitative observations defining the levels of intestinal IL-18 in both germline and somatic NLRC4-AID with and without MAS could help elucidate the origin of IL-18 in human NLRC4-AID.

Another qualitative difference potentially contributing to the lack of MAS in our NLRC4-AID patients is the normal levels of IL-12p70. IL-12p70 and IL-18 act in synergy to stimulate IFN-γ production [18], and IL-12 neutralization ameliorates disease in an IFN-γ-dependent mouse model of MAS [19, 20]. Furthermore, systemic co-administration of IL-18 and IL-12, but not either cytokine alone, induced IFN-γ-dependent intestinal inflammation in mice [21]. Altogether, these data suggest that IL-12p70 can modulate both systemic and intestinal IFN-γ-dependent inflammation, at least in mice. In the context of normal IL-12p70 levels, IL-18 alone might be unable to induce sufficient IFN-γ production to drive MAS. The role of IL-12p70 in human MAS has not yet been clearly defined.

A defect in cell-mediated cytotoxicity underlies primary HLH by preventing proper termination of immune responses [15]. Differences in natural killer (NK) cell function could therefore be another qualitative factor influencing MAS susceptibility. Various studies on NK cells in SD and MAS have reported contradictory results, and a transient NK cell dysfunction induced by the continuous inflammatory environment, partially attributable to chronic IL-18 exposure, has been proposed [22]. There is currently no data available on NK cell cytotoxic function and cytokine production in NLRC4-AID, and its relevance for MAS development in this context remains unexplored.

It remains noteworthy that the highest levels of free IL-18 in the somatic NLRC4-AID patient were detected during treatment with canakinumab. The lack of MAS in this patient could therefore be partially explained by ongoing IL-1β inhibition.

The detection of free IL-18 in NLRC4-AID and *PSTPIP1*-mutated PAPA [16] patients without MAS point towards a pathological role of IL-18 beyond MAS. Indeed, despite IL-1β or TNF-α inhibition, ESR is seldomly in the normal range in our FCAS4 and somatic NLRC4-AID patients even during periods of clinical remission (data not shown). It could be argued that the observed residual inflammation is, at least partly, due to bioactive free IL-18. These findings provide a rationale for therapeutically targeting IL-18 in both germline and somatic NLRC4-AID without MAS.

The results presented herein are hampered by small sample size and genetic homogeneity. Future studies would benefit from international collaboration to increase both sample size and the number of included NLRC4 variants. Despite these limitations, our findings add yet another layer of complexity to IL-18 biology and our understanding of NLRC4-AID.

## Supplementary Material

keag363_Supplementary_Data

## Data Availability

The data that support the findings of this study are available from the corresponding author upon reasonable request.
