# Supplementary material for: Free IL-18 in NLRC4-associated autoinflammatory disease without macrophage activation syndrome
Source: Rheumatology (Oxford). 2026 Jul 13;65(7):keag363. doi: 10.1093/rheumatology/keag363 (PMC13395081; doi:10.1093/rheumatology/keag363)
Supplement: keag363_Supplementary_Data [file keag363_supplementary_data.docx]

Supplementary material

Supplementary Figure S1: correlation between total and free-IL-18 and laboratory markers of inflammation and MAS in NLRC4-AID patients without MAS.


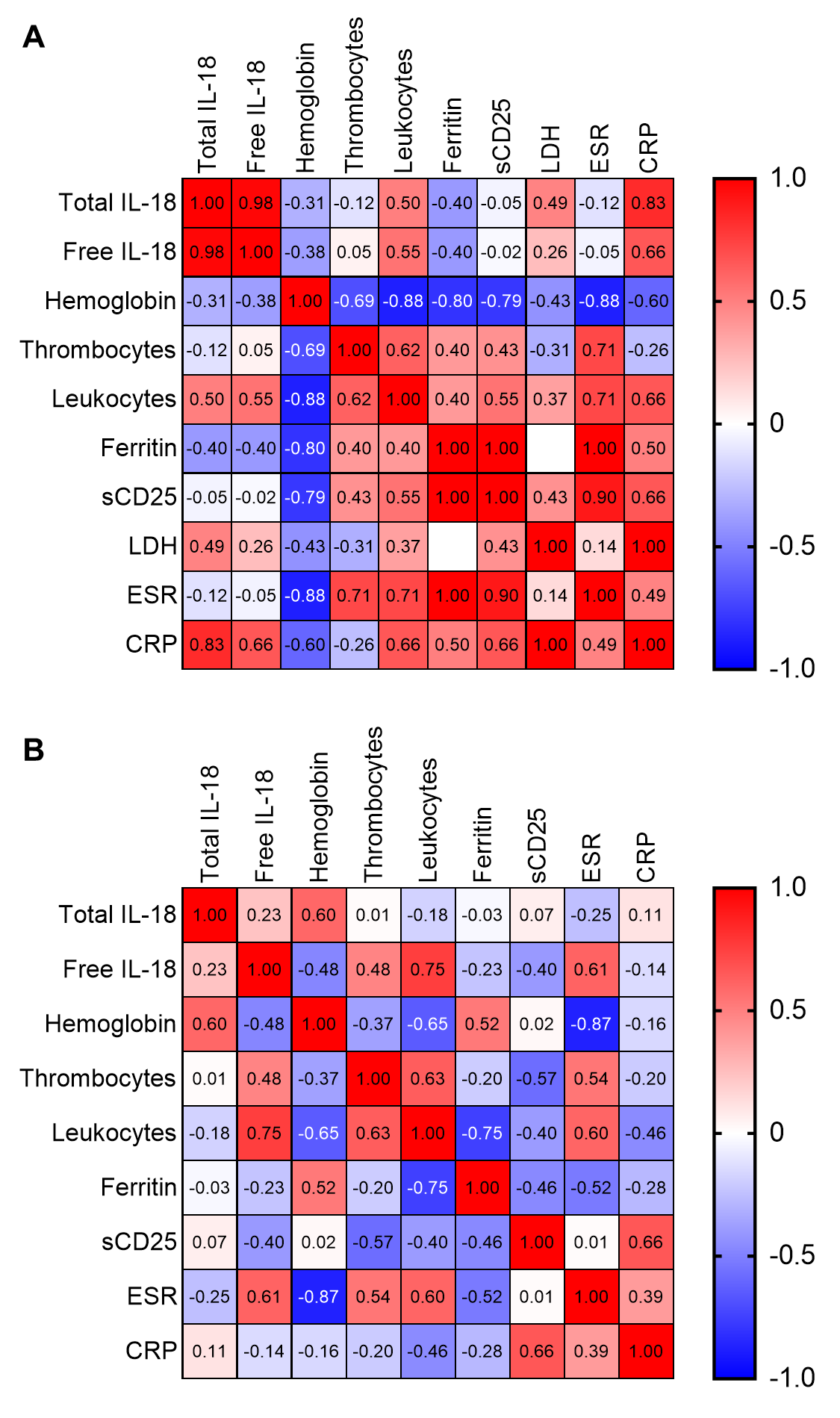


Intensity plot of Spearman correlation table in longitudinal samples from a somatic NLRC4-AID patient (A) and 5 FCAS4 patients (B). Spearman correlation was not calculated for LDH in FCAS4 patients due to low variability.

Supplementary Figure S2: Temporal relationship between total and free IL-18 and laboratory markers of inflammation and MAS in a somatic NLRC4-AID patient.


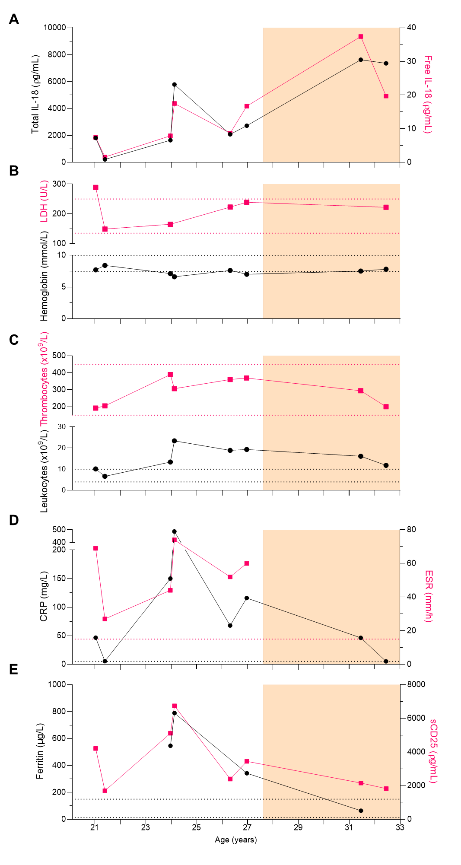


Longitudinal samples of total and free IL-18 (A), hemoglobin and LDH (B), leukocytes and thrombocytes (C), CRP and ESR (D) and ferritin and sCD25 (E). Orange background represents treatment with canakinumab. Dotted lines represent reference value or range.

Supplementary Figure S3: Levels of TNF-α, IL-1β IL-12p70 and IL-1Ra in NLRC4-AID without MAS.


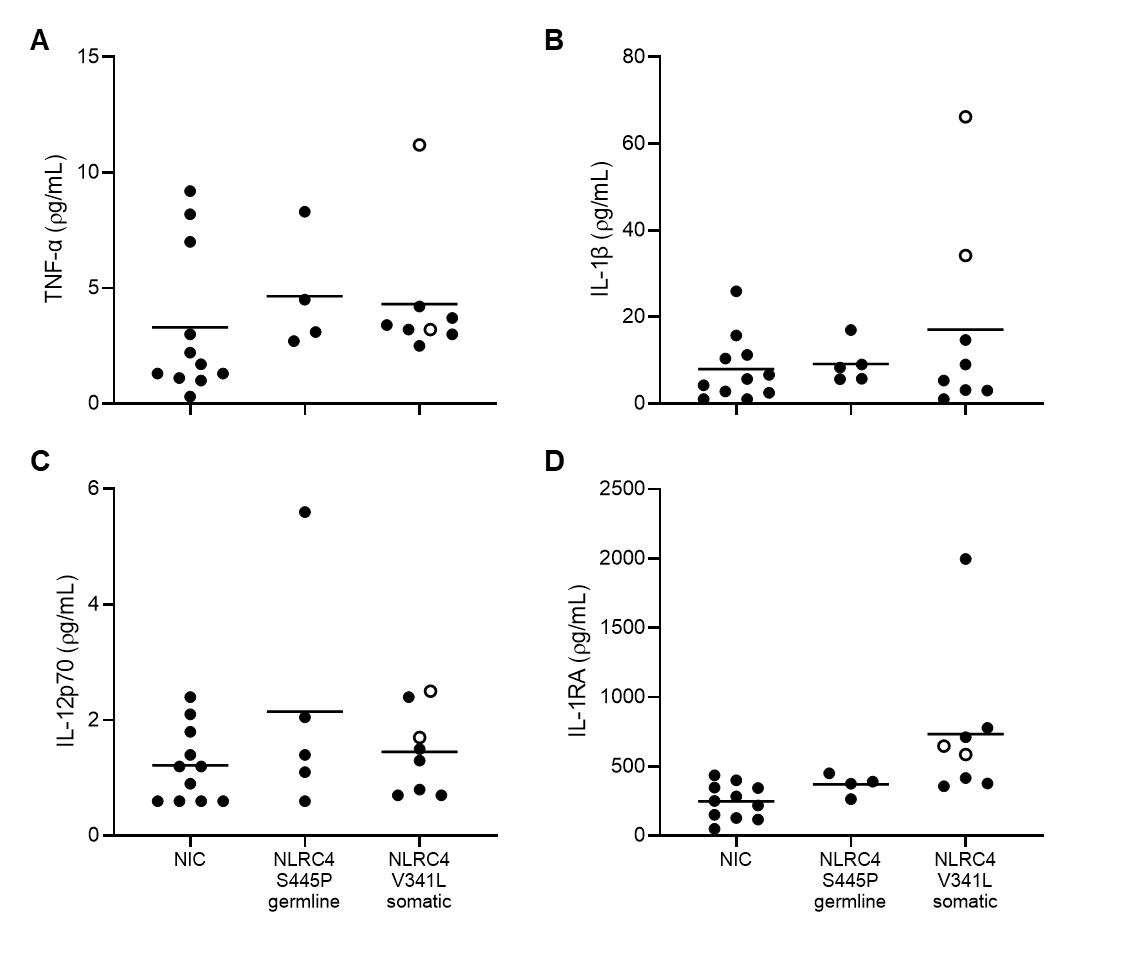


Levels of TNF-α (A), IL-1β (B), IL-12p70 (C) and IL-1Ra (D) in NIC, FCAS4 and a somatic NLRC4-AID patient. For the somatic NLRC4-AID patient, open circles depict samples taken during treatment with canakinumab. For TNF-α, 4 samples from 3 FCAS4 patients were excluded from statistical analysis as they were taken during treatment with etanercept, which is known to increase/stabilize TNF-α. For IL-1Ra, 2 samples from 2 FCAS4 patients were excluded from statistical analysis as they were taken during treatment with anakinra.

Supplementary Table S1: Clinical and laboratory characteristics of FCAS4 patients

|  | **Gender** | **Age** | **Current treatment** | **Hb** | **Thrombo** | **Leuko** | **NK cells** | **ESR** | **CRP** | **Ferritin** | **sCD25** | **Fibrino** | **Triglyc** | **LDH** |
| --- | --- | --- | --- | --- | --- | --- | --- | --- | --- | --- | --- | --- | --- | --- |
| **Reference range** |  |  |  | 7.3-11.0 mmol/L | 130-350 ·10^9^/L | 3.5-11.0 ·10^9^/L | 65-423 cells/µL | <19 mm/h | ≤10 mg/L | 15-400 µg/L | <600 U/mL | 1.7-4.0 g/L | 0.80-1.94 mmol/L | <248 U/L |
| Fresh samples | | | | | | | | | | | | | | |
| P1 | F | 58 | Etanercept | 8.6 | 253 | 5.7 | *52* | *31* | 8 | 43 | 356 | 3.6 | 1.32 | *275* |
| P2 | F | 36 | Etanercept | 8.7 | 213 | 5.2 | 105 | *32* | *27* | 45 | 487 | *4.2* | *2.89* | *266* |
| P3 | F | 55 | Anakinra (first dose) | 7.6 | 255 | 5.5 | 355 | *37* | *12* | 79 | 332 | *4.4* | 1.02 | *265* |
| P4 | M | 66 | Etanercept | *7.2* | 223 | 6.4 | 230 | *59* | *12* | 43 | 417 | 3.8 | 1.85 | *260* |
| P5 | F | 64 | Self-requested drug holiday | 7.4 | *380* | 8.4 | 205 | *99* | *18* | 37 | 352 | *5.6* | 1.65 | *256* |
| Historical samples | | | | | | | | | | | ρg/mL |  |  |  |
| P1 | F | 48 | Etanercept | 9.4 | 252 | 5.7 |  | *25* | 3 | 48 | 2240 |  |  |  |
| P2 | F | 31 | None | 7.8 | 252 | 5.3 |  | *37* | *43* |  | 3805 |  |  |  |
| P4 | M | 62 | Anakinra |  |  |  |  |  |  |  | 2453 |  |  |  |
| P5 | F | 59 | None | 7.3 | *362* | 10.0 |  | *60* | *11* |  | 2256 |  |  |  |

Hb : hemoglobin, Thrombo: thrombocytes, Leuko: leukocytes, NK cells: natural killer cells, ESR: erythrocyte sedimentation rate, CRP: C-reactive protein, sCD25: soluble CD25, Fibrino: fibrinogen, Triglyc: triglycerides, LDH: lactate dehydrogenase. Values outside the reference range are depicted in italic.

Supplementary Table S2: Clinical and laboratory characteristics of the somatic NLRC4-AID patient

| **Age** | **Treatment** | **Hb** | **Thrombo** | **Leuko** | **ESR** | **CRP** | **Ferritin** | **sCD25** | **LDH** |
| --- | --- | --- | --- | --- | --- | --- | --- | --- | --- |
| **Reference range** |  | 7.4-9.9 mmol/L | 150-450  ·10^9^/L | 4.0-10.0 ·10^9^/L | <15  mm/h | ≤5 mg/L | 13-150  µg/L | ρg/mL | 135-250  U/L |
| 21 | Ibuprofen | 7.7 | 192 | *10.1* | *69* | *47* |  | 4207 | *289* |
| 21 | Ibuprofen | 8.4 | 205 | 6.6 | *27* | *6* |  | 1695 | 149 |
| 23 | Ibuprofen | *7.1* | 389 | *13.4* | *44* | *150* | *546* | 5111 | 165 |
| 24 | Ibuprofen | *6.6* | 306 | *23.4* | *74* | *487* | *789* | 6737 |  |
| 26 | Methylpred  AZA | 7.6 | 360 | *18.9* | *52* | *68* |  | 2391 | 223 |
| 27 | Methylpred  AZA | *7.0* | 369 | *19.3* | *60* | *116* | *342* | 3443 | 239 |
| 31 | Methylpred  Canakinumab | 7.5 | 294 | *16.1* |  | *46* | 64 | 2156 |  |
| 32 | Methylpred  Canakinumab | 7.8 | 200 | *11.8* |  | 5 |  | 1821 | 222 |

Hb : hemoglobin, Thrombo: thrombocytes, Leuko: leukocytes, ESR: erythrocyte sedimentation rate, CRP: C-reactive protein, sCD25: soluble CD25, LDH: lactate dehydrogenase, methylpred: methylprednisolone, AZA
